# Vulnerability in adolescents’ daily life during the COVID-19 pandemic

**DOI:** 10.1590/1980-220X-REEUSP-2023-0100en

**Published:** 2023-10-13

**Authors:** Aline Cammarano Ribeiro, Fabiano Ritta Malagues Ianzer, Fernanda Duarte Siqueira, Eliane Tatsch Neves, Neila Santini de Souza, Camila Nunes Barreto, Graciela Dutra Senhem, Cíntia Vanuza Monteiro Bugs

**Affiliations:** 1 Universidade Federal de Santa Maria, Santa Maria, RS, Brazil.; 2 Universidade Federal de Santa Maria, Palmeira das Missões, RS, Brazil.; 3 Universidade Luterana do Brasil, Cachoeira do Sul, RS, Brazil

**Keywords:** Adolescent, COVID-19, Health Vulnerability, Adolescente, COVID-19, Vulnerabilidad en Salud, Adolescente, COVID-19, Vulnerabilidade em Saúde

## Abstract

**Objective:**

To know the vulnerability in adolescents’ daily life during the COVID-19 pandemic.

**Method:**

Qualitative research carried out with 25 adolescents from a public school in a municipality in southern Brazil in the second half of 2021 through individual semi-structured interviews. Data were submitted to thematic content analysis and interpreted with the vulnerability theoretical framework.

**Results:**

These adolescents’ daily lives during the COVID-19 pandemic presented difficulties in keeping family members working, changing habits and routines, accessing classes, the internet and increasing intra-family violence.

**Conclusion:**

Vulnerability in adolescents’ daily life during the pandemic can be identified as the absence of interaction in the school context and access to learning resources, reflecting on individual and social vulnerability. Unemployment and possible access to other sources of income have an impact on programmatic vulnerability. Reflection on practices in the context of health and school is suggested, based on the vulnerability identified in adolescents’ daily lives during the COVID-19 pandemic.

## RESUMO

**Objetivo:**

Conhecer a vulnerabilidade no cotidiano de adolescentes na pandemia de COVID-19.

**Método:**

Pesquisa qualitativa, realizada com 25 adolescentes de uma escola pública em um município do Sul do Brasil, no segundo semestre de 2021, por meio de entrevistas semiestruturadas individuais. Os dados foram submetidos à análise temática de conteúdo e interpretados com o referencial teórico da vulnerabilidade.

**Resultados:**

O cotidiano desses adolescentes na pandemia de COVID-19 apresentou-se com dificuldades na manutenção de empregos dos familiares, mudança de hábitos e rotinas, forma de acesso às aulas, internet e aumento da violência intrafamiliar.

**Conclusão:**

A vulnerabilidade no cotidiano de adolescentes durante a pandemia pode ser identificada como a ausência do convívio no contexto escolar e acesso aos recursos para aprendizagem, refletindo na vulnerabilidade individual e social. O desemprego e o possível acesso de outras fontes de renda repercutem na vulnerabilidade programática. Sugere-se a reflexão sobre práticas no contexto da saúde e da escola, tendo por base a vulnerabilidade identificada no cotidiano de adolescentes na pandemia de COVID-19.

## RESUMEN

**Objetivo:**

Conocer la vulnerabilidad en el cotidiano de los adolescentes ante la pandemia de COVID-19.

**Método:**

Investigación cualitativa, realizada con 25 adolescentes de una escuela pública de un municipio del sur de Brasil, en el segundo semestre de 2021, a través de entrevistas semiestructuradas individuales. Los datos fueron sometidos al análisis de contenido temático e interpretados con el referencial teórico de la vulnerabilidad.

**Resultados:**

El cotidiano de estos adolescentes durante la pandemia de la COVID-19 presentó dificultades para conservar el trabajo de los familiares, cambio de hábitos y rutinas, acceso a clases, internet y aumento de la violencia intrafamiliar.

**Conclusión:**

La vulnerabilidad en el cotidiano de los adolescentes durante la pandemia se puede identificar como la ausencia de interacción en el contexto escolar y acceso a los recursos de aprendizaje, reflexionando sobre la vulnerabilidad individual y social. El desempleo y el posible acceso a otras fuentes de ingresos inciden en la vulnerabilidad programática. Se sugiere la reflexión sobre las prácticas en el contexto de la salud y la escuela, a partir de la vulnerabilidad identificada en el cotidiano de los adolescentes en la pandemia de la COVID-19.

## INTRODUCTION

COVID-19 is a disease caused by SARS-CoV-2 that ­emerged at the end of 2019 in Wuhan, China, and was declared by the World Health Organization (WHO) as a public health emergency, being designated as a pandemic. Health measures to control contagion to ensure the protection of the population, slow down the growing trend of transmission and prevent the collapse of health services are elements that can change people’s daily lives. This disease affects individuals of all ages, socioeconomic status, sex and ethnicity^(1)^. Until the end of the 34^(2)^. Between July 1, 2021 and December 31, there were 3,730,380 cases registered. In September, the Ministry of Health recommended the vaccination of adolescents aged 12 to 17 years^(3)^.

In view of this, as a result of the COVID-19 pandemic, in March 2020 the Ministry of Education decreed, as of Ordinance 343^(4)^, the suspension of on-site classes and their consequent replacement by online activities anchored in digital means, and in this situation, schools were closed. Furthermore, the Brazilian National Health Council, under Recommendation 036 of May 11, 2020^(5)^, recommended isolation and social distancing measures to prevent and control the coronavirus, with the suspension of all non-essential activities for maintenance of life and health, with restrictions on the movement of people.

In view of this, adolescents and their families needed to readapt their routine, learning mode and financial resources. In this way, the separation of adolescents from friends, teachers, loved ones, loss of freedom and uncertainty about the disease occurred. Given the magnitude of this circumstance and understanding that adolescence is a moment that goes through different biological, psychological and social transformations, this may constitute a long-term problem in these adolescents’ lives^(6,7)^.

When facing a new disease, it is important to consider the daily life of each person, the meanings attributed to the disease, the risks, culture, access to prevention, care and treatment^(8)^. Daily life is a term that comes from the Latin *cotidie* or *cotidianus*, which means everyday, the daily, the common and the usual, which refers to thinking directly about actions that relate to routines and everything that is done empirically repeatedly, living day to day^(9)^. In this way, adolescents present a daily life before the pandemic and during the pandemic, the latter with changes resulting from the new forms imposed by the virus.

Based on data published in a scoping review by Brazilian authors, aimed at adolescent health in times of COVID-19, the need for further studies on this topic was identified^(10)^. Studies with adolescents during the COVID-19 pandemic address mental health issues and violence. Violence against women, children and adolescents in times of a COVID-19 pandemic is configured with the need for coping actions, due to the increase in the occurrence of violence^(11)^. Another study shows that as a result of the pandemic, with the impact of social distancing, there were cases of underreporting in the identification of violence against children and adolescents^(12)^. In addition to this, studies address the negative impact on adolescent mental health, associated with sudden interruptions in habits and routines resulting from the pandemic^(13-14)^.

It is known that, in situations of disasters/pandemics, vulnerability emerges in children’s and adolescents’ daily lives. Vulnerability can be defined as an expanded and reflective perception that discusses the exposure of individuals to injuries and their consequent impacts involving individual, social and programmatic components. The individual refers to the degree and quality of information that individuals have about the problem and the possibility of using it for protection. The social involves aspects that depend on access to the means of communication, availability of material resources and political factors. The programmatic is related to the actions of programs aimed at prevention and care, which may be regional, local and national policies that must be made available in an effective and democratic way. Therefore, the components occur in an integrated manner, due to greater or lesser access to resources of all orders^(15)^.

This research, based on the vulnerability theoretical framework^(15)^, can support nursing, health and education professionals to develop practices that address the challenges arising from adolescence they experience, added to a pandemic of social, economic, individual, political and emotional scope. Therefore, the present study aimed to know the vulnerability in adolescents’ daily lives during the COVID-19 pandemic.

## METHOD

### Study Design

This is qualitative research^(16)^ of descriptive and exploratory type. The COnsolidated criteria for REporting Qualitative research (COREQ)^(17)^ checklist criteria were followed in conducting and preparing the research report.

### Study Setting

The study took place in a state elementary school, from the 1^st^ to the 9^th^ grades, located in the north of the municipality of Santa Maria, Rio Grande do Sul, Brazil. It has a total of 203 students enrolled, 74 students from the initial grades from the 1^st^ to the 4^th^ grades and 129 from the 5^th^ to the 9^th^ grades, with 18 teachers working. Part of these students live in situations of vulnerability, some of which include precarious housing conditions, difficulties in maintaining basic needs and unemployment in the family. This setting was intentionally chosen, because in this school, extensionist health promotion actions are developed with school adolescents, coordinated by the guiding teacher of this study, which enabled a prior relationship between the research team and participants.

### Population and Selection Criteria

The participants who were part of this study were students enrolled in that school. Regarding the selection criteria, adolescents aged between 10 and 19 years, according to the WHO^(18)^, regularly enrolled and attending school at the time of data collection were included. Thus, the selection of study participants took place through intentional sampling^(19)^. All adolescents invited to participate in the study were recommended by the school’s professional teachers. Thus, adolescents from the 5^th^ to the 9^th^ grades who fit the age group and were interested in participating participated. The 9th grade students were invited but did not want to participate, and did not report any justification.

### Data Collection

The approach with the study setting took place through contact with a professional reference teacher with greater proximity and bond with school students. From this, a conversation was scheduled with teachers and school management, moment when the study proposal, the importance and process of how the interviews with adolescents would be conducted were presented. Soon after, a draft was sent to school management, containing the main information that would be collected from adolescents. Then, a list of the names of students in the school’s classes was made available to the researchers. It should be noted that, during this period, the school was in the process of returning to on-site classes, from Monday to Friday. But, at first, it was optional for students to be present in the classroom, and they could remain with remote classes if they wished. At that time, the school was adjusting to the return of classes. After that, students who were present in the class were contacted, one class at a time, collectively, and the adolescents were invited and explained about the research. Upon invitation, those interested in participating in the study were given the Informed Consent Form (ICF), for signature at the home by parents or legal guardian, and the Assent Form (AF), for minors under 18 years old, emphasizing that after accepting and signing the terms, students should deliver them to the school and then a shift and time was scheduled, according to student availability, to carry out the individual interview at school. Data collection took place between August and September 2021.

For data collection, a sociodemographic characterization form of adolescents was initially used, containing the following variables: sex, age, grade and how many people live in the house. Subsequently, individual semi-structured interviews were carried out by the main researcher on-site in an available space within the school provided by the institution, respecting and guaranteeing participants’ privacy. The interviews were guided by issues involving daily life, family routine, school activities, distancing from friends, colleagues, family, leisure activities during the pandemic. A pre-test was carried out with adolescents linked to the members of the research group for quality of writing and understanding in order to minimize errors in data collection. There were changes in relation to the formulation of the questions, making them increasingly closer to adolescents. Biosafety and SARS-CoV-2 prevention measures were followed at the time of data collection. The interviews were recorded in audio, with authorization, using a digital recorder. Later, they were transcribed in full and had an average duration of 30 minutes.

### Data Analysis and Treatment

The interviews were transcribed, organized and subjected to thematic content analysis, consisting of the following steps: a) pre-analysis, in which adolescents’ testimonies were skimmed through, initiating direct and intense contact with the field material; b) material exploration, guided by the exhaustive reading of the material, seeking to find meaningful categories according to which the content of one speech or more will be organized; and c) data treatment and interpretation, with the theoretical framework initially designed, or opening tracks around new theoretical and/or interpretative dimensions^(15)^. In this way, the thematic category in which the results are presented was defined.

### Ethical Aspects

The study followed the ethical aspects of research with human beings, according to Resolution 466/2012 of the Brazilian National Health Council, which deals with the Guidelines and Regulations for Research Involving Human Beings. The study was approved by the Research Ethics Committee (REC) under Registration 4,823,496 in 2021. Prior to the interview, the ethical questions provided included knowledge and signature of the ICF by adolescents over 18 years of age, but this did not occur, as these invited adolescents did not want to participate. Adolescents under 18 years of age were introduced to the AF, and to guardians, the ICF. From the signing of the terms, data collection began. As a guarantee of participant anonymity, they were identified by the letter “A”, referring to Adolescent, followed by an Arabic numeral according to the order of the interviews (A1, A2...A10...A25).

## RESULTS

A total of 25 adolescents participated in this study, 13 female and 12 male, aged between 11 and 15 years, attending the 5^th^ to 8^th^ grades. Eleven adolescents lived with their stepmother or stepfather and siblings, and sometimes grandparents, uncles and nephews also lived together. Another nine adolescents lived with their father, mother and siblings; two adolescents, with mother, grandparents, uncles, cousins, brothers, nephews and brother-in-law; an adolescent, with grandparents and siblings; and two adolescents, with uncles and cousins.

From the analysis of results, the following thematic category was identified: The different faces of the COVID-19 pandemic and vulnerability in adolescents’ daily life.

### The Different Faces of the Covid-19 Pandemic and Vulnerability in Adolescents’ Daily Life

The COVID-19 pandemic triggered different faces in ­adolescents’ daily lives. To this end, the implications of the ­pandemic on family relationships were signaled in their speeches, which were sometimes demonstrated with more unity, presence, approach of parents, conversations and, on the other hand, the fights decreased, as shown in the following reports:

*The father and mother were more absent, and now they are a little more present. (A3)*

*The father and mother grew closer during the pandemic. (A18)*

*The good thing is that everyone stayed together at home, family united at home, happy, no one could leave. (A20)*

*My stepfather and mother fought a lot before the pandemic. One good thing that happened was that the fights decreased [...] we got together more, we spent more time together. (A21)*

*It was peaceful, the family seems to be more united, we spend more time together. (A24)*

*I stayed at home watching TV with the family [mother and father], watching videos, playing video games, it was calmer [...] it was good, the family was more united, there was more conversation. (A25)*

In adolescents’ daily lives during the COVID-19 ­pandemic, they mentioned changes such as job losses, moving house, mother’s depression, need for adolescents to do some work to help at home, which was very bad according to adolescents, triggering anxiety about families’ financial health, signaling a programmatic vulnerability according to the following statements:

*My mom lost her job and so did my stepdad and then we had to move in with my aunt. (A5)*

*My mother did the cleaning and with the pandemic she didn’t have it anymore. She has a depression crisis and it got stronger, and I end up having to take care of her a little because it’s just me. (A14)*

*It was bad, my aunt had to stop working, so only my uncle worked, I helped when I could, I did odd jobs (I work as a freelancer). (A15)*

*My stepfather, my two brothers and my cousin lost their jobs in the pandemic. My mother and sister also had to stop working during this COVID, so that was bad. (A19)*

*It was a little difficult because my mother works doing nails and most people didn’t go, then my mother did not earn money, but luckily my father is working. (A20)*

Regarding the school, adolescents mentioned that school activities were developed through reading materials and exercises available via Classroom, which is a school content management system, or printed at school. Adolescents referred to difficulties in participating in online classes and handling technology. Other adolescents brought limitations on access to available resources (cell phone, computer, internet), which influenced learning, and they also reported that they could not learn properly due to the accumulation of school activities. Faced with these difficulties, at times they had the help of family members, but they considered that teaching with the teacher was better. Some adolescents missed school, because of their relationships with peers and because it is a place they like to be, in addition to the fact that learning occurs more effectively, making it more difficult to learn separately from school, constituting individual, social and programmatic vulnerability according to the following narratives:

*One difficulty was that I had to stop going to school, which for me was always a corner that I liked [...] we did it online, and the teachers left material for us to go to school to get the activities and we did it. When we didn’t know, we did it through the Classroom, and I asked my sister or my uncle for help. (A4)*

*I had no internet, no cell phone, so my mother had to pick up the material at school. It was difficult because it’s better with the teacher than with my mother. (A5)*

*I didn’t like the online classes, I thought it was boring because I couldn’t learn properly, it was very bad, there was a lot of accumulated things to do, and the activities were bad for me because I didn’t know how to do the activities. (A6)*

*The education was bad because it’s not the same as going to an on-site class. (A10)*

*I missed school, I couldn’t talk to my classmates [...], I cry because there’s no way to go to school, much better at school, my writing is very ugly, it’s still horrible, it’s harder for me to separate from school it got even worse, I stopped doing things. (A19)*

*I thought it was difficult because we didn’t have a computer and the cell phone belonged to my aunt, it was complicated for me, I liked it best when the school was open so I could do the activities and that’s it. (A23)*

During the pandemic, adolescents reported that they stopped carrying out some activities that were part of their daily lives, especially with regard to leisure, such as playing ball in the street, hanging out with friends and family, visiting. Now, they only stayed at home, based on the recommendations made by the Brazilian National Health Council for isolation and social distancing, thus revealing a social vulnerability, according to the following statements:

*I used to play soccer in the street with the people I knew and now it’s not possible anymore because their parents won’t let them out. (A1)*

*Not being able to leave the house, I didn’t talk to people much anymore and then I ended up being more isolated, the pandemic took all of that away and even left us without friends. (A6)*

*I invited the aunts out and together we used to go to the square to talk, walk around and now I couldn’t anymore, I had to stay at home more. (A7)*

*I don’t go out much like I used to see my friends, having to stay at home and not being able to go out is a struggle, but it’s strange not being able to visit relatives as usual. (A12)*

*I couldn’t play football with my friends and also hang out with my mother. (A14)*

*I couldn’t go out so it was bad because I liked to go play with friends, talk and now I’m just at home it’s boring. (A17)*

Some adolescents reported situations of physical violence and self-mutilation. Self-mutilations were frequent; suicide attempt occurred; and intrafamily violence was caused by the stepfather or brother-in-law, reported by adolescents, which increased during the pandemic period, which represents an individual social vulnerability, according to the following reports:

*I cut myself a lot and I tried to kill myself too, I went to the hospital because I took hidden medicine, I didn’t cut myself before the pandemic, I didn’t care about anything, but now I don’t know, I think I got more fragile. Before I was quite strong, I didn’t care, I was fine, minding my own business, not now. (A2)*

*At the beginning of the pandemic, I cut my arms and legs with a razor and now I kind of look at the videos on the internet and try not to do it anymore, I kind of feel a pain in my heart, I feel alone. (A4)*

*My stepfather beat me more during the pandemic, he stayed at home more. My father found out and he took custody out of my mother. (A11)*

*My brother-in-law is leaving the house because he ended up hitting my sister and then she doesn’t want to be with him anymore. (A14)*

*My stepdad started hitting my mom and I walked over to him and started hitting him, he stopped and apologized to me. (A19)*

Faced with reports of violence and mutilations, support was provided and support and comfort were offered by the ­researchers and assistance in seeking psychological treatment, circumstance in which comfort was the support that adolescents most desired at that moment.

## DISCUSSION

The data produced by adolescents showed that there were difficulties in maintaining jobs for family members, changing habits and routines, in the form of access to classes, the internet and the increase in intra-family violence in their daily lives during the COVID-19 pandemic. Furthermore, it provided greater interaction between adolescents and their families at home. Therefore, these factors influenced the exposure of adolescents to individual, social and programmatic vulnerability.

Regarding adolescents’ greater family life, the pandemic positively facilitated the union, but sometimes reinforced conflicts, which implies social vulnerability. This converges with a study carried out with adolescents in northeastern Brazil, which brought the family together to face the pandemic, in which it showed integrating and disintegrating family relationships^(20)^. Another study showed that the pandemic triggered greater interaction between the adolescent and the family, which may represent protection for adolescents from possible illnesses^(20)^. This protection can be made possible through communication between parents and children, with the possibility of helping children’s and adolescents’ mental health, mitigating situations of anxiety and depression^(21).^

During the pandemic, social distancing ended up becoming the main way to contain the speed of contamination by COVID-19^(5)^, producing negative economic consequences such as a decrease in people’s income, in which companies fired employees with a formal contract and informal workers lost their livelihood^(22)^. In view of this, the health measures established to contain the COVID-19 pandemic resulted in precarious labor relations, reduced working hours, among others. This precariousness of the economic conditions of adolescents and their respective families increases the social vulnerability that the pandemic has imposed on these people’s daily lives.

According to data from the United Nations International Children’s Emergency Fund (UNICEF), families with children and adolescents aged 0 to 17 reported that their income was affected by the pandemic, with the need to resort to other sources of income such as federal, state and municipal government benefit, family allowance, food donation, unemployment insurance and Continuing Provision Benefit^(23)^. Unemployment and possible access to other sources of income are also related to programmatic vulnerability^(15)^, in which there is a need for actions, programs and policies that effectively subsidize dignified conditions to live. A study carried out in the United States with adolescents during the pandemic pointed out that financial difficulties and pandemic-related stress served as risk factors, while parental coping and involvement served as protective factors, associated with a drop in school ties^(24)^.

Adolescents’ quality of life and teaching were impacted by COVID-19. This concerns programmatic and social vulnerability, as adolescents, for their development, need access to the means of communication, availability of material resources and from these accesses seek quality information and develop it for their protection^(15)^.

During the COVID-19 pandemic, families had to readjust and reconcile their children’s education and work according to their conditions experienced by social distancing. Education during the pandemic was carried out in an improvised way, causing losses in learning, and it is necessary to restore this damage by offering support to families, adolescents and educators^(25)^. A study carried out in Turkey showed that adolescents’ satisfaction with school life was impaired due to the uncertainties they faced during the COVID-19 pandemic associated with high levels of stress^(26)^. Responsibility for the education of children initially refers to the family and, in this context, the school predisposes to a teaching-learning environment. Several parents do not have pedagogical training to mediate contents of subjects of specific knowledge. In addition to their work demands, or lack of them, they feel overwhelmed in monitoring their children’s school activities^(27)^.

Still in the school context, there are the adolescents’ affective relationships, in which it is often in these relationships that they find support to experience the phase of adolescence. A study carried out in China pointed out that adolescent friendships can be significantly associated with their psychological well-being^(28)^. During the pandemic, there were impediments to broad social contacts and limited coexistence between people, making traditional ways of experiencing leisure unfeasible, such as family parties, meetings with friends, team sports and the possibility of traveling^(29)^.

With social distancing, people who had access to technologies found in virtual leisure a way to distract themselves and adapt their routines, through digital and social media. Paradoxically, there has been a significant increase in the use of the internet. With this, the use of technology has skyrocketed, such as the use of applications, social networks, television subscriptions, among others, which showed that people had the human need to maintain their social relationships by enjoying the technological environment as leisure^(28)^.

Also referring to relationships, in some adolescents’ daily lives there was intra-family tension and stress, due to the intensification of family life in addition to anxieties, fear and economic and social crisis. A study carried out with adolescents that investigated the impact of COVID-19 on children’s and adolescents’ mental health showed that it was generally negative, in which conflicts increased in the family environment^(21)^. It should be noted that the family can also be an integrator in the context of pandemic changes with adolescents, developing family care based on trust and protection^(20)^.

In relation to situations of intrafamily violence and self-mutilation, with the changes in coexistence, they became more frequent and intense or originated in this period. During social isolation, the home was at risk of becoming a very dangerous place, with domestic violence occurring, due to the recommendation to stay at home. The routine of being with family members prevented people from being close to friends and significant others^(30)^. A study that analyzed the rates of reports of violence against children and adolescents in the state of Rio Grande do Sul during the period of the COVID-19 pandemic found that there was a decrease in the rates of reports of violence against children and adolescents, requiring actions to improve the recognition of cases suspected of violence during the pandemic^(12)^.

A limitation of this study is that it was carried out in only one school, characterizing research at the local level, not representing different contexts, which could possibly imply different daily lives and vulnerability. Due to the restrictions and extreme precautions resulting from the pandemic, it was decided to do it in a school, which the research team already had prior ­contact with.

The contributions of this study are directed towards the visibility of the vulnerability of adolescents’ daily life during the COVID-19 pandemic, and that their individual, social and programmatic dimensions are recognized and discussed.

## CONCLUSION

From the study carried out, it was possible to know the vulnerability present in adolescents’ daily lives during the COVID-19 pandemic. It was observed in the social dimension the absence of school life and learning, such as access to classes, internet and resources and communication devices available to continue studies. Moreover, during the pandemic period, the effects on adolescents’ health were intense, as they were confined to their homes, without outdoor activities and interaction with friends and peers. For some adolescents, the presence of domestic violence occurred, which weakened the coexistence between family members.

Involving the individual and programmatic dimensions, the lack of access to technology and connectivity faced by adolescents who do not have or have not been able to develop skills in the use of these new technological tools was highlighted. Unemployment and possible access to other sources of income were related to the programmatic component. However, the components of vulnerability are present in adolescents’ daily life during the pandemic, as living is multifaceted and happens in an interconnected way, and may be more or less susceptible to a certain component. Reflection on practices in the context of health and school is suggested, based on the vulnerability identified in adolescents’ daily lives during the COVID-19 pandemic.

## Data Availability

https://doi.org/10.48331/scielodata.ISC0UC
